# Out-of-Hospital Excess Mortality From Cardiovascular Diseases During the COVID-19 Pandemic in Brazil

**DOI:** 10.7759/cureus.108136

**Published:** 2026-05-02

**Authors:** Humberto G Moreira, Thiago C Barreto

**Affiliations:** 1 Medical School, Federal University of Goias, Goiania, BRA

**Keywords:** cardiovascular death, covid-19, excess mortality, out-of-hospital, public health system

## Abstract

Background: The COVID-19 pandemic disrupted health systems worldwide; yet, the magnitude of collateral mortality occurring outside hospitals, particularly from cardiovascular disease (CVD), remains poorly characterized in low- and middle-income countries (LMICs). Brazil, which experienced one of the highest cumulative death tolls globally, offers a critical setting for quantifying this indirect burden.

Methods: We conducted an ecological time-series analysis of all deaths recorded outside health care facilities (domicile or public thoroughfare) in Brazil from 2015 through 2021 using national mortality data from the Mortality Information System (SIM)/Department of Informatics of the Brazilian Unified Health System (DATASUS). Crude mortality rates and age-sex adjusted mortality rates were calculated and standardized to the 2022 Census population structure. Pre-pandemic (2015-2019) and pandemic (2020-2021) periods were compared descriptively.

Results: Out-of-hospital deaths increased from a pre-pandemic mean of 313660 per year (151.6 per 100000 inhabitants) to 381720 per year (180.2 per 100000 inhabitants) during 2020-2021, representing an 18.8% increase. Age-sex adjusted cardiovascular out-of-hospital mortality rates, which had declined steadily from 53.43 per 100000 in 2015 to 47.16 per 100 000 in 2019, rose sharply to 53.48 per 100000 in 2020 and 54.53 per 100000 in 2021. The Southeast exhibited the steepest rate increase (+23%), whereas the Northeast maintained the highest overall proportion of out-of-hospital cardiovascular deaths. CVD remained the leading cause of death (115228; 29.8%), followed by external causes (65009; 16.8%) and ill-defined causes (62433; 16.1%). Ill-defined deaths increased by 44% between 2019 and 2021, suggesting substantial diagnostic gaps.

Conclusions: The pandemic period was associated with a substantial rise in out-of-hospital mortality in Brazil, reversing the prior declining trend in age-sex adjusted cardiovascular mortality. These findings underscore the need for resilient primary care networks and decentralized emergency response systems during public health emergencies.

## Introduction

The severe acute respiratory syndrome coronavirus 2 (SARS-CoV-2) pandemic imposed an unprecedented strain on health care systems worldwide. Hospitals redirected resources toward intensive care expansion, suspended elective procedures, curtailed outpatient follow-up, and redeployed clinical personnel to emergency and critical care services [1,2]. Simultaneously, public health authorities implemented social distancing mandates, stay-at-home orders, and restrictions on non-essential services. Although these measures were essential for limiting viral transmission, they also generated an unintended consequence: populations with chronic diseases, particularly cardiovascular conditions, experienced reduced access to the very care systems designed to prevent acute decompensation and death [3,4].

Emerging evidence from high-income countries documented a striking rise in out-of-hospital cardiac arrests (OHCA) during the early pandemic period. In New York City, non-traumatic OHCA increased threefold in 2020 compared with 2019, peaking at 305 daily cases on April 6, 2020, nearly a tenfold increase relative to the same date in the preceding year [5]. In Italy, OHCA incidence rose by 58%, predominantly involving non-shockable rhythms and substantially reduced rates of return of spontaneous circulation [6]. Population-based data from the Greater Paris area similarly demonstrated a near doubling of weekly OHCA incidence, from 13.4 to 26.6 per million inhabitants [7].

Evidence from low- and middle-income countries (LMICs), however, remains substantially more limited despite the disproportionate mortality burden borne by these settings. The World Health Organization estimated that 86% of pandemic excess mortality occurred in LMICs. In Ecuador, cardiometabolic deaths accounted for 30.4% of all excess mortality in 2020, with acute myocardial infarction showing the greatest relative increase [8]. In Peru, geospatial analyses of the national death registry revealed significant increases in cardiovascular mortality during pandemic waves [9]. The CARDIO COVID-19-20 Registry, encompassing 3260 patients from 14 Latin American countries, documented high rates of in-hospital cardiovascular complications [10]. A systematic review of excess mortality in low- and lower-middle-income countries found that observed deaths exceeded expected levels by approximately 65% [11]. Yet, cause-specific analyses of out-of-hospital mortality, particularly cardiovascular deaths occurring without contact with health care systems, remain limited in LMIC settings, thereby limiting the evidence base for targeted preparedness strategies.

Despite this growing body of evidence, a critical gap persists. Most published analyses focus exclusively on the first pandemic wave in 2020 and originate predominantly from high-income countries, with limited representation from LMICs that bore the greatest mortality burden. Brazil, a continental nation of over 210 million people marked by profound regional disparities in health care access, socioeconomic indicators, and epidemiological profiles, offers a uniquely informative case. Moreover, the Brazilian pandemic trajectory differed meaningfully from that of European nations and the United States: the country experienced its most devastating mortality in 2021, marked by the predominant circulation of the Delta variant, when 424107 COVID-19 deaths were recorded alongside 14.6 million confirmed cases [12]. This period, often referred to as the "second wave" in the Brazilian context, was characterized by unprecedented strain on the health care infrastructure. Accordingly, any analysis restricted to 2020 alone would substantially underestimate the indirect burden of the pandemic in this setting.

The Brazilian Mortality Information System (SIM), linked to the national Department of Informatics of the Brazilian Unified Health System (DATASUS) platform, provides a comprehensive population-level registry of all registered deaths, classified by ICD-10 codes and location of occurrence. This infrastructure enables the systematic examination of out-of-hospital mortality, defined as deaths occurring at the decedent’s domicile or on public thoroughfares without contact with health care services, across the entire national territory and over multiple years.

In this study, we leveraged this national dataset to characterize the magnitude, temporal trajectory, regional distribution, and cause-of-death profile of out-of-hospital mortality in Brazil during the pre-pandemic quinquennium (2015-2019) and the pandemic biennium (2020-2021). Our primary objectives were threefold: (1) to describe changes in out-of-hospital mortality between the pre-pandemic and pandemic periods; (2) to identify the leading causes of death occurring outside health facilities and their relative changes; and (3) to examine regional heterogeneity as a proxy for structural inequities in health system resilience.

## Materials and methods

Study design and setting

We conducted an ecological time-series study using secondary mortality data. The unit of analysis comprised all out-of-hospital deaths registered in Brazil between January 1, 2015, and December 31, 2021, aggregated at the national and macro-regional levels. The study period was divided into a pre-pandemic reference period (2015-2019) and a pandemic period (2020-2021).

Data sources

Mortality data were extracted from the SIM, managed by the Department of Health Information Analysis within the Brazilian Ministry of Health's Secretariat of Health Surveillance and accessed through the DATASUS public-access platform [13]. Death certificates in this system are coded according to the International Classification of Diseases, Tenth Revision (ICD-10). Data were extracted in January 2026 using the database version dated December 2, 2025. Population denominators were obtained from the Brazilian Institute of Geography and Statistics (IBGE). Mortality rates were recalculated using population estimates adjusted to the 2022 National Census, which provided more accurate denominators than the 2010-based intercensal projections previously available.

Case definition and inclusion criteria

Out-of-hospital deaths were defined as all deaths with the place of occurrence coded as domicile or public thoroughfare (via pública) in the SIM database, excluding deaths in hospitals, health care facilities, or during emergency medical service transport. This classification follows the standard coding categories of the Brazilian death certificate (Declaração de Óbito). All registered deaths meeting this criterion during the study period were included, irrespective of age, sex, or underlying cause.

Variables and stratification

Deaths were classified by ICD-10 chapter-level cause-of-death groupings and stratified by Brazilian geographic macro-region (North, Northeast, Southeast, South, and Central-West). We separately tabulated deaths coded as ill-defined and unknown causes of mortality (ICD-10 Chapter XVIII: R00-R99), given the epidemiological significance of diagnostic uncertainty during periods of health system strain.

Statistical analysis

Crude mortality rates were calculated as the number of out-of-hospital deaths per 100000 inhabitants per year. Age-sex adjusted mortality rates for cardiovascular out-of-hospital deaths were calculated by direct standardization using the 2022 Census population as the reference, stratified by sex and five-year age groups. This adjustment was performed to ensure temporal comparability of rates across years with different population structures. The objective was to generate comparable annual rates, not to explore age- or sex-specific mortality differences among individuals who died outside health services.

Pre-pandemic period rates were computed as the annual mean for 2015-2019. The percentage change in mortality rate between the pre-pandemic and pandemic periods was calculated as: Percentage change (%)= ((Rate_pandemic_-Rate_pre-pandemic_)/Rate_pre-pandemic_)x100.

Given the census-derived, population-level nature of the dataset, which encompasses all registered deaths rather than a probability sample, conventional inferential statistics (confidence intervals, hypothesis testing) were not applied. All estimates represent descriptive population parameters.

All data were presented as absolute counts and rates; comparisons were descriptive, given the population-level, census-derived nature of the dataset.

Ethical considerations

This study used exclusively de-identified, publicly available secondary data from the SIM/DATASUS platform. Institutional review board approval was not required under Brazilian regulatory norms (Resolution CNS No. 510/2016) for research conducted solely with publicly accessible aggregate data.

## Results

Temporal trends in out-of-hospital mortality

Between 2015 and 2019, a total of 6553132 deaths were registered in Brazil, of which 1568298 (23.9%) occurred outside health care facilities, at the decedent's domicile or on public thoroughfares. During the pandemic biennium (2020-2021), total registered deaths rose to 3383178, with 763440 (22.6%) classified as out-of-hospital. The mean annual count of out-of-hospital deaths increased from 313660 during the pre-pandemic period to 381720 during 2020-2021, representing a relative increase of 21.7% (n=68060 additional deaths per year). In 2021 alone, out-of-hospital deaths (n=401128) exceeded the 2019 figure (n=326047) by 23.0%.

**Figure 1:**
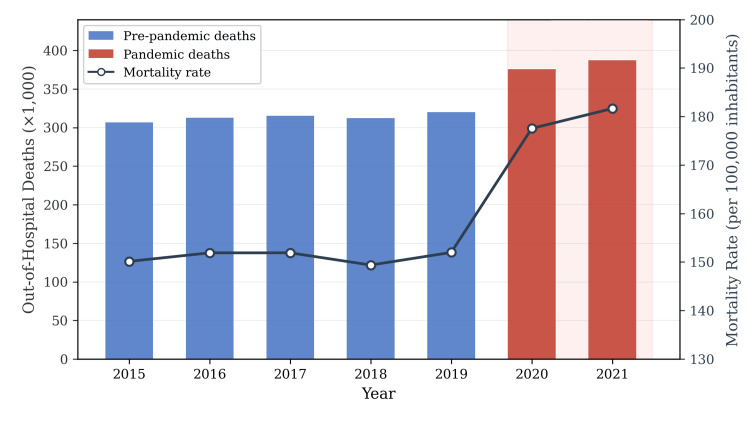
Temporal trends in out-of-hospital deaths (bars, left axis) and crude mortality rate per 100000 inhabitants (line, right axis) in Brazil, 2015-2021 Blue bars represent the pre-pandemic reference period (2015-2019), and red bars denote the pandemic biennium (2020-2021). The annotation indicates the 23.0% increase in absolute out-of-hospital deaths between 2019 and 2021. Data source: SIM/DATASUS. SIM: Mortality Information System; DATASUS: Department of Informatics of the Brazilian Unified Health System

The crude out-of-hospital mortality rate rose from 151.6 per 100000 inhabitants (pre-pandemic mean) to 180.2 per 100000 inhabitants during 2020-2021, representing an absolute increase of 28.6 deaths per 100000 and a relative increase of 18.8%. Notably, the pre-pandemic period (2015-2019) exhibited relative stability in both absolute counts and mortality rates, followed by a sharp inflection in 2020 that steepened further in 2021 (Figure 1).

**Figure 2:**
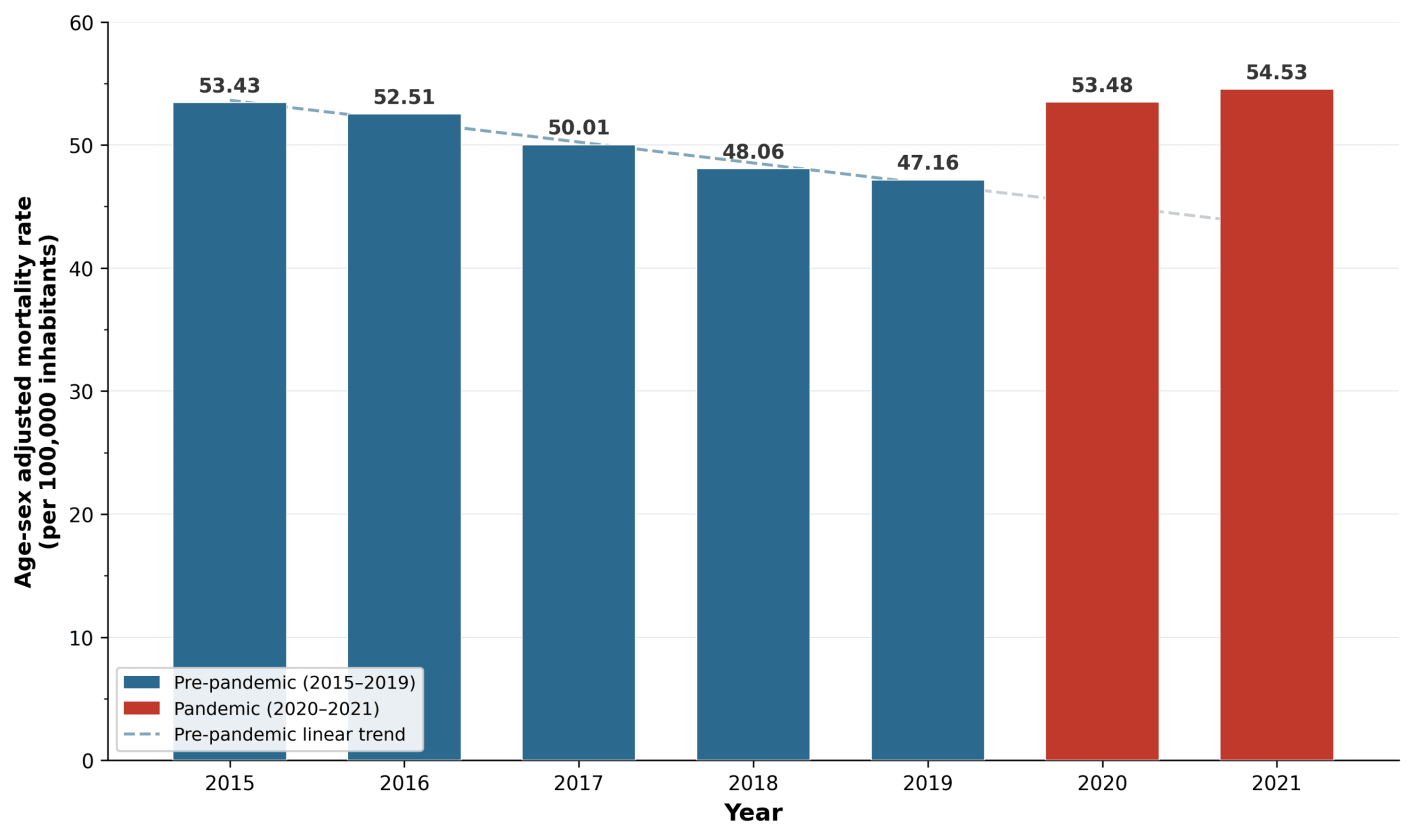
Adjusted out-of-hospital cardiovascular mortality Age-sex adjusted out-of-hospital cardiovascular mortality rate per 100000 inhabitants in Brazil, 2015-2021. Rates were standardized by the direct method using the 2022 Census population. Blue bars represent the pre-pandemic reference period (2015-2019), and red bars denote the pandemic biennium (2020-2021). The dashed line indicates the pre-pandemic linear trend. Data source: SIM/DATASUS; population denominator: IBGE/2022 Census. SIM: Mortality Information System; IBGE: Brazilian Institute of Geography and Statistics; DATASUS: Department of Informatics of the Brazilian Unified Health System

Age-sex adjusted cardiovascular out-of-hospital mortality rates confirmed and strengthened this pattern (Figure 2). The adjusted rate had been declining consistently during the pre-pandemic period, from 53.43 per 100000 inhabitants in 2015 to 47.16 per 100000 in 2019, representing an 11.7% reduction over five years, consistent with the secular trend of improving cardiovascular outcomes in Brazil. This declining trajectory was sharply reversed during the pandemic: the adjusted rate rose to 53.48 per 100000 in 2020 and 54.53 per 100000 in 2021, surpassing the 2015 baseline. The 2021 adjusted rate exceeded the 2019 value by 15.6% (from 47.16 to 54.53 per 100000). These findings demonstrate that the observed increase in out-of-hospital cardiovascular mortality was not attributable to demographic shifts alone but represented a genuine epidemiological inflection.

Regional heterogeneity

The pandemic-related surge in out-of-hospital mortality was observed across all five macro-regions, though its magnitude varied substantially. The Southeast region, home to the largest population and most urbanized centers, exhibited the steepest relative increase in both absolute deaths (+26%) and mortality rate (+23%). The North region followed, with a 19.4% increase in mortality rate. By contrast, the Northeast, despite recording the highest absolute proportion of out-of-hospital deaths and the highest overall mortality rate (232 per 100000 inhabitants), showed the smallest relative increase (+15% in mortality rate and +17% in absolute deaths).

Cause-of-death profile

Diseases of the circulatory system remained the predominant cause of out-of-hospital death throughout the study period, accounting for 115228 (29.8%) of all such deaths in 2021. External causes (injuries, violence, and accidents) constituted 65009 (16.8%), ill-defined and unknown causes 62433 (16.1%), and neoplasms 43261 (11.2%). A comparative analysis of cause-specific trends between the pre-pandemic and pandemic periods revealed a heterogeneous pattern of change (Table 1).

**Table 1:** Out-of-hospital deaths by ICD-10 cause-of-death chapter in Brazil, 2015-2021 ^*^Mortality rate per 100000 inhabitants. Δ 2019-2021 denotes the percentage change between 2019 and 2021 mortality rates. Data source: SIM/DATASUS. ICD: International Classification of Diseases; SIM: Mortality Information System; DATASUS: Department of Informatics of the Brazilian Unified Health System

Cause of death (ICD-10)	2015		2016		2017		2018		2019		2020		2021		Δ 2019-2021
	Deaths (n)	Rate^*^	Deaths (n)	Rate^*^	Deaths (n)	Rate^*^	Deaths (n)	Rate^*^	Deaths (n)	Rate^*^	Deaths (n)	Rate^*^	Deaths (n)	Rate^*^	
Circulatory system diseases	96908	47.40	98628	47.80	97299	46.90	9694	46.50	9864	46.90	112306	53.00	115228	54.00	15.10%
External causes	70947	34.70	71358	34.60	7305	35.20	67311	32.30	62183	29.60	65809	31.10	65009	30.50	3.00%
Ill-defined causes	43134	21.10	44776	21.70	41818	20.10	3947	18.90	43375	20.60	56824	26.80	62433	29.30	42.20%
Neoplasms	31092	15.20	3161	15.30	33404	16.10	34169	16.40	34987	16.60	4423	20.90	43261	20.30	22.30%
Endocrine/metabolic	19156	9.40	19546	9.50	20196	9.70	20752	10.00	21772	10.40	29431	13.90	29749	13.90	33.70%
Respiratory system	18321	9.00	1894	9.20	19631	9.50	18802	9.00	1899	9.00	18946	8.90	17103	8.00	-11.1%
Nervous system	9181	4.50	9664	4.70	10327	5.00	11111	5.30	12694	6.00	16536	7.80	1725	8.10	35.00%
Mental/behavioral disorders	5286	2.60	5146	2.50	5401	2.60	5713	2.70	6117	2.90	9052	4.30	9285	4.40	51.70%
Infectious/parasitic	4146	2.00	4402	2.10	4399	2.10	4165	2.00	4126	2.00	11413	5.40	14689	6.90	245.00%
Digestive system	635	3.10	6406	3.10	6213	3.00	6252	3.00	6209	3.00	7289	3.40	7309	3.40	13.30%
Genitourinary system	2499	1.20	2577	1.25	2754	1.30	292	1.40	321	1.50	4203	2.00	4001	1.90	26.70%

Cardiovascular deaths occurring outside hospitals increased by 16.8% between 2019 and 2021 (from 98640 to 115228; n=16588 additional deaths), and neoplasm-related out-of-hospital deaths rose by 23.6% (from 34987 to 43261; n=8274). The corresponding mortality rates showed a similar pattern (Table 1). However, the most dramatic relative increases were observed in categories indicative of diagnostic gaps and systemic disruption. Deaths attributed to infectious and parasitic diseases surged by 255.9% (from 4126 in 2019 to 14689 in 2021; n=10563 additional deaths). This increase was driven predominantly by COVID-19 deaths occurring outside health care facilities: nationally, 6258 out-of-hospital COVID-19 deaths were recorded in 2020 and 9771 in 2021. Full disaggregation of COVID-19-specific out-of-hospital deaths requires individual-level SIM microdata and is planned for subsequent analysis. Deaths classified as ill-defined causes increased by 44.0% between 2019 and 2021 (from 43375 to 62433; n=19058), representing a signal of reduced diagnostic capacity during periods of overwhelming system strain. Deaths due to mental and behavioral disorders rose by 51.8% (from 6117 in 2019 to 9285 in 2021; n=3168).

## Discussion

This population-level analysis of over 2.3 million out-of-hospital deaths in Brazil during 2015-2021 describes a substantial and sustained rise in mortality occurring outside health care facilities during the COVID-19 pandemic. The 18.8% increase in the out-of-hospital mortality rate during 2020-2021, with an even sharper 23% surge in 2021 compared with 2019, is consistent with the interpretation that the pandemic's lethal impact extended far beyond its direct viral toll. These findings position Brazil within a global pattern of pandemic-associated indirect mortality while highlighting features unique to a large, heterogeneous middle-income country.

Diseases of the circulatory system accounted for nearly one-third of all out-of-hospital deaths during the pandemic (115228; 29.8% in 2021), with a 16.8% increase relative to 2019. This finding is consistent with and extends observations from high-income settings. In New York City, Lai et al. documented a threefold increase in non-traumatic OHCA in 2020, disproportionately affecting individuals of advanced age, non-White race/ethnicity, and those with hypertension or diabetes [5]. Baldi et al. reported a 58% rise in OHCA in Lombardy, with a striking predominance of non-shockable rhythms, suggesting that many patients presented only after irreversible myocardial damage had occurred [6].

Several pathophysiological mechanisms may contribute to the observed excess cardiovascular mortality. SARS-CoV-2 infection has been associated with systemic inflammation, endothelial dysfunction, and a prothrombotic state that may precipitate acute cardiovascular events [7,14-17]. However, these mechanisms were not directly measured in our ecological study. At the population level, the observed increase in out-of-hospital cardiovascular deaths likely reflects a combination of direct viral effects in undiagnosed individuals and the consequences of health care system disruption in dealing with a poorly understood and highly transmissible disease.

Although health-seeking avoidance was not directly measured in our dataset, external evidence supports this interpretation. De Filippo et al. documented reductions in acute myocardial infarction admissions of up to 40.2% in northern Italy, and Guimarães et al. reported a 33% increase in domiciliary cardiac arrest deaths in Belo Horizonte [18,19]. These observations, together with our finding of increased out-of-hospital cardiovascular mortality, are consistent with the hypothesis that avoidance of emergency care may have contributed to excess domiciliary deaths.

The 44.0% increase in deaths attributed to ill-defined and unknown causes between 2019 and 2021 (from 43375 to 62433; n=19058 additional deaths) may serve as a proxy for reduced diagnostic capacity at the point of death. The surge in ill-defined mortality during the pandemic may reflect a convergence of factors, including overwhelmed death investigation systems, reduced availability of medical death certifiers, and undiagnosed COVID-19 deaths occurring before testing could be performed [20]. While this pattern is consistent with reduced diagnostic capacity, alternative explanations cannot be excluded.

This diagnostic gap represents not merely a statistical artifact but also a potential threat to epidemiological surveillance. Accurate cause-of-death coding is the foundation upon which public health resource allocation, disease burden estimation, and pandemic response evaluation rest.

The regional analysis reveals a notable pattern. The Northeast, Brazil's most socioeconomically disadvantaged macro-region, maintained the highest absolute out-of-hospital mortality rate (232 per 100000 inhabitants) throughout both the pre-pandemic and pandemic periods, yet exhibited the smallest relative increase during the crisis (+15%). Conversely, the Southeast, with its more developed health infrastructure, registered the steepest relative surge (+23%). This pattern may reflect, in part, pre-existing structural determinants such as poverty, limited health care access, and historically higher rates of ill-defined death coding, which may have constrained the capacity for relative increases during acute crises [11,21]. However, this interpretation is speculative, and alternative explanations, including differential underreporting, cannot be excluded.

Marinho et al. documented racial disparities in excess mortality within São Paulo during the pandemic [21]. Marinelli et al. further noted that the Northeast's role as a major tourist destination may have facilitated early SARS-CoV-2 dissemination, with severe outcomes in states such as Piauí and Pernambuco [22].

The 51.8% increase in out-of-hospital deaths attributed to mental and behavioral disorders (from 6117 in 2019 to 9285 in 2021; n=3168 additional deaths) warrants particular attention. Although the absolute numbers are modest relative to cardiovascular mortality, this category captures deaths from substance use disorders, dementia-related complications, and other conditions exacerbated by social isolation, loss of support networks, and psychological distress. Pereira-Ávila et al. documented depressive symptoms among Brazilian older adults during the early pandemic, suggesting that mental health effects were present; however, the causal pathway from distress to excess mortality requires individual-level investigation [23].

The age-sex adjusted analysis provides additional confidence in these findings. The pre-pandemic declining trend in cardiovascular out-of-hospital mortality, from 53.43 to 47.16 per 100000 between 2015 and 2019, was consistent with secular improvements in cardiovascular care and prevention in Brazil. The sharp reversal to 54.53 per 100000 in 2021 indicates that the observed increase cannot be attributed to demographic aging alone and instead represents a genuine disruption of the favorable trajectory established over the preceding quinquennium.

Our findings are consistent with emerging LMIC evidence. In Ecuador, cardiometabolic deaths accounted for 30.4% of excess mortality in 2020 [8]. The COVID-19 MORtality (C-MOR) consortium documented variable cause-specific excess mortality across 12 countries with different health system capacities [24]. A systematic review of excess mortality in low- and lower-middle-income countries found that observed deaths exceeded expected levels by approximately 65% [11]. These parallels suggest that the cardiovascular burden of the pandemic may represent a shared feature across LMICs.

The Brazilian data extend the international literature in three important ways. First, by encompassing the full 2020-2021 biennium rather than the first pandemic wave alone, our analysis captures the sustained and, in many respects, worsening trajectory of out-of-hospital mortality. Second, the inclusion of all-cause out-of-hospital deaths, rather than only cardiac arrests, provides a more comprehensive picture of the indirect burden. Third, the regional stratification highlights how structural inequities modulate the impact of a global health crisis, producing divergent mortality patterns even within a single national health system.

Limitations

This study has several limitations. First, the ecological design analyzes aggregated population-level data and does not permit causal inference; the observed associations between the pandemic period and increased out-of-hospital mortality, while temporally coherent, may be confounded by secular trends or reporting artifacts. Second, cause-of-death coding in national mortality registries is subject to misclassification, a concern amplified during the pandemic when diagnostic and death-certification processes were under extraordinary strain; the increase in ill-defined causes of death itself reflects this vulnerability. Third, although age-sex adjusted rates were calculated to ensure temporal comparability, detailed stratification by age group, sex, or comorbidity burden was not performed because it was beyond the scope of this descriptive analysis. Fourth, misclassification of the place of death may have occurred. Deaths occurring during EMS transport or at the threshold of emergency departments may have been variably coded as hospital or other, and such misclassification may have changed during the pandemic when emergency departments were overwhelmed. Fifth, the aggregate data format did not permit full disaggregation of COVID-19-specific deaths within the infectious and parasitic disease category. Finally, SIM data quality varies across Brazilian states, with historically higher rates of under-registration in the North and Northeast.

## Conclusions

The COVID-19 pandemic period was associated with a marked increase in out-of-hospital mortality across Brazil during 2020-2021, reversing the prior declining trend in age-sex adjusted cardiovascular mortality rates. Regional disparities are consistent with the amplifying effect of pre-existing structural inequities.

In the Brazilian context, these findings highlight the importance of maintaining continuity of primary care, including chronic disease follow-up through community health agents and telemedicine. Pre-hospital emergency networks require contingency protocols for surge scenarios, and cause-of-death surveillance systems require investment in medical death certification training and integration of point-of-care testing for out-of-hospital death investigation. Further studies using individual-level data are needed to refine these findings.
